# Acute Myelomonocytic Leukemia: A Rare Cause of Painless Jaundice

**DOI:** 10.7759/cureus.77223

**Published:** 2025-01-10

**Authors:** Amey Joshi, Rohan Kumar, Ankit Kulkarni, Ryan K Mui, Adarsh Jha, Tadd K Hiatt

**Affiliations:** 1 Internal Medicine, Michigan State University, Lansing, USA; 2 Gastroenterology, Michigan State University, Lansing, USA; 3 Gastroenterology, University of Michigan, Lansing, USA

**Keywords:** acute myeloid leukemia (aml-m6), cholestasis, jaundice cholestatic, new-onset painless jaundice, thrombocytopenia

## Abstract

Acute myelomonocytic leukemia (AMML) is a rare and aggressive subtype of acute myeloid leukemia with varying clinical manifestations. Although hepatic involvement in AMML is commonly seen, cholestatic hepatitis as a primary manifestation of the disease is very rare. In the present case, our elderly patient presented with predominant signs of cholestasis, including jaundice, nausea, and vomiting. However, after a thorough diagnostic workup that ruled out obstructive, viral, or drug-induced etiologies, the presence of severe thrombocytopenia prompted further investigation. Flow cytometry was ultimately utilized to diagnose our patient with AMML. This case highlights that laboratory evidence of hematological abnormalities, including thrombocytopenia, could aid in diagnosing unexplained cholestasis outside the recommended workup.

## Introduction

Acute myelomonocytic leukemia (AMML) is a rare and aggressive subtype of acute myeloid leukemia (AML). Early diagnosis of AMML can be challenging due to its variable presentation [1]. Although hepatic involvement is observed in AMML, the occurrence of cholestatic jaundice as a primary manifestation is rarely reported [2]. Various etiologies have been identified to explain cholestatic liver injury in AMML, including leukemic infiltration of the hepatic sinusoids, hemophagocytic lymphohistiocytosis (HLH), and drug-induced liver injury [3-5]. We present a patient with clinical signs of cholestasis without radiological evidence of biliary obstruction and with severe thrombocytopenia. The thorough evaluation of her thrombocytopenia ultimately led to the new diagnosis of AMML. This case highlights the challenges of the diagnosis of AMML in the offsetting of extramedullary manifestation of cholestasis.

## Case presentation

A 62-year-old female presented with complaints of jaundice, nausea, vomiting, diarrhea, subjective fevers, and general malaise of one-week duration. Her history was notable for chronic lower back pain, previously treated with 1200 mg ibuprofen daily, which was recently substituted for acetaminophen daily due to new-onset lower abdominal cramping. She denied any melena, hematochezia, hematemesis, weight changes, other medication or supplement use, recent illness, recent travel, alcohol intake, or family history of liver disease.

**Figure 1:**
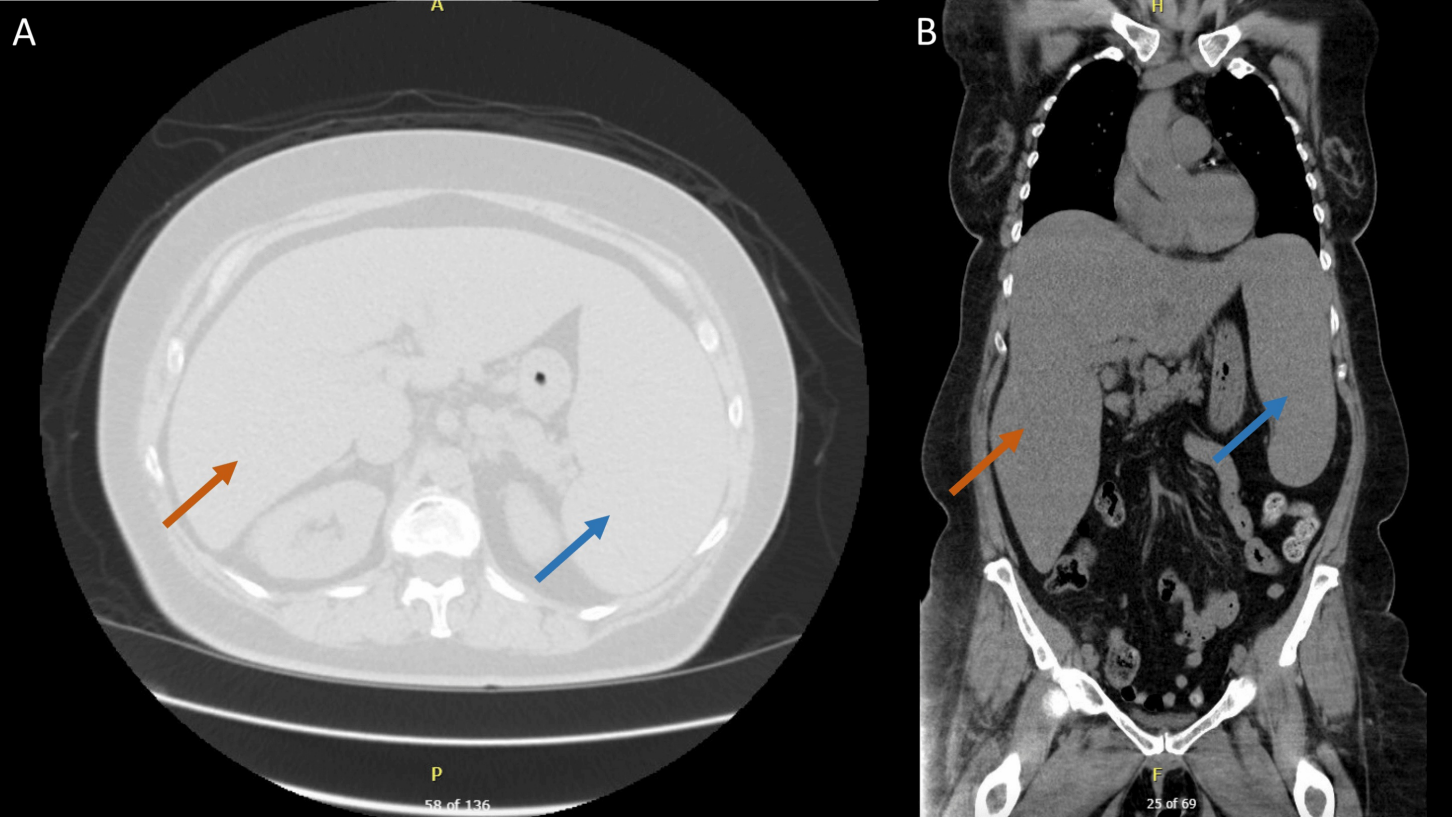
CT abdomen showing hepatomegaly (red arrow) and splenomegaly (blue arrow) A: axial section; B: coronal section

Physical examination was significant for icterus, generalized abdominal tenderness, and an erythematous rash in her bilateral lower extremities. Vitals were unremarkable. Laboratory investigation was noteworthy for mild transaminitis (aspartate transaminase 62 IU/ml, alanine transaminase 73 IU/ml), elevated alkaline phosphatase (455 U/L), conjugated bilirubinemia (total bilirubin 14.8, conjugated bilirubin 14.5), a prothrombin time of 12.4 seconds, leukocytosis (22.2 x 10^3 u/L), severe thrombocytopenia (12 x 10^3 u/L), and creatinine and blood urea nitrogen (BUN) of 2.83 mg/dl and 18 ml/min, respectively (Table 1). Abdominal ultrasound was suggestive of hepatic steatosis with hepatomegaly but was negative for any gallbladder or common bile duct abnormalities. CT abdomen was done, and hepatosplenomegaly was confirmed (Figure 1). Non-steroidal anti-inflammatory drug (NSAID)-related liver injury was suspected; however, labs did not improve despite NSAID discontinuation after seven days of last use.

**Table 1:** Laboratory investigations on admission AST: aspartate transaminase; ALT: alanine transaminase; ALP: alkaline phosphatase; BUN: blood urea nitrogen

Lab Test	Lab Value on Admission	Reference Range
White Blood Cell Count (WBC)	22.2/mm^3^	4.5 - 11/mm^3^
Neutrophils	2 %	49-81 %
Lymphocytes	10 %	14-41 %
Monocytes	86 %	0-11 %
Myelocytes	1 %	
Metamyelocytes	1 %	
Red Blood Cell Count (RBC)	2.26 million/mm^3^	3.5 - 5.5 million/mm^3^
Hemoglobin (Hgb)	12.3 g/dL	12 - 16 g/dL
Hematocrit (Hct)	36.3%	36% - 46%
Mean Corpuscular Volume (MCV)	84 µm³	80 - 100 µm³
Platelets (Plt)	12/mm^3^	150 - 400/mm^3^
Total Bilirubin	14.8 mg/dL	0.1 - 1.0 mg/dL
Direct Bilirubin	14.5 mg/dL	0.0 - 0.3 mg/dL
AST	62 U/L	10-40 U/L
ALT	73 U/L	3-45 U/L
ALP	455 U/L	50-130 U/L
Creatinine	2.83 mg/dl	0.60-1.40 mg/dl
BUN	43 mg/dl	6-23 mg/dl

Flow cytometry was then used to further evaluate severe thrombocytopenia, which revealed absolute monocytosis with immature monocytes, most consistent with acute myelomonocytic leukemia (AMML). It was deemed that the patient’s current cholestatic disease was secondary to an infiltrative process related to her AMML. Having discussed the guarded prognosis in this condition, the patient declined to pursue further investigations with bone marrow biopsy or aggressive treatment with chemotherapy. Serial investigations revealed worsening total bilirubin (15.6). After a thoughtful discussion of her goals of care, the patient transitioned to hospice care and peacefully passed at home a week after her discharge.

## Discussion

The etiology of conjugated hyperbilirubinemia can broadly be classified by hepatocellular injury or cholestasis. Cholestasis can be categorized as caused by intrahepatic cholestasis, most commonly seen in drug-induced injury, infiltrative disease, and sepsis; and biliary obstruction, as seen in choledocholithiasis and obstructive tumors. The workup of these etiologies primarily involves the assessment of liver enzymes (AST, ALT, and ALP) and imaging through ultrasound or magnetic resonance cholangiopancreatography [6]. AML is a rare cause of cholestatic liver injury, with only a handful of cases reported [7]. As a result, a hematological workup is not commonly indicated in cholestasis. In this case, the presence of thrombocytopenia led to the diagnosis of AMML through flow cytometry. AMML is a rare subtype of AML with considerable overlap in evaluation and management. As most evidence of cholestasis in the present topic is derived from cases of AML, this discussion will highlight relevant pathophysiology, workup, and treatment options.

AML can cause cholestatic liver injury through a variety of different mechanisms. Leukemic cell infiltration into the liver, resulting in granulocytic sarcomas, has been found to cause bile duct obstruction, leading to jaundice [3]. AML has also been associated with the formation of biliary strictures, another common cause of cholestasis [4]. The inflammatory milieu in secondary HLH in AML can cause direct liver damage and cholestasis through cytokine and macrophage activation [5]. Cytarabine, commonly used during induction and maintenance therapy in AML, can also cause cholestasis through direct hepatocellular injury [8]. Although these mechanisms and associations have been identified, a complete workup for cholestasis is crucial before initiation of chemotherapy.

Conjugated hyperbilirubinemia, as mentioned earlier, can be caused by hepatocellular injury or cholestasis. Liver enzyme levels (AST, ALT, and ALP) are commonly assessed to differentiate hepatocellular from cholestatic injury, wherein a higher AST and ALT level than ALP indicates a hepatocellular injury. A higher ALP and gamma-glutamyl transferase (GGT) level than AST and ALT are more likely to indicate a cholestatic picture. The R-factor is a standard calculated method utilized to differentiate between these two etiologies, wherein an R-factor of less than two and more than five indicates a cholestatic picture and hepatocellular picture, respectively. In the present case, the R-factor was calculated to be 0.5, indicative of cholestasis. Ultrasound or magnetic resonance cholangiopancreatography (MRCP) is indicated in the event of a cholestatic liver injury to assess for bile duct dilation or stricture [6]. Abdominal ultrasound in our patient did not reveal any bile duct abnormalities that suggested a purely obstructive cause of cholestasis. The presence of severe thrombocytopenia in the present case prompted further evaluation through flow cytometry, which led to the diagnosis of AML. Acute myeloid leukemia (AML) should be considered as a cause of jaundice in patients who have an associated cytopenia within days or weeks, along with the presence of circulating blast cells in the peripheral blood. These blasts display a high nuclear-to-cytoplasmic ratio, irregular nuclear contours, and smooth chromatin with prominent or multiple nucleoli. A liver biopsy is usually considered if liver dysfunction is pronounced and other non-leukemic causes have been ruled out. Genetic and molecular studies help identify specific genetic mutations associated with AMML, which may further guide prognostication and therapeutic intervention [9].

The outcome of AML remains poor, with an overall survival of 35-60%. Extramedullary manifestations in AML further confer a poorer prognosis overall [10]. The so-called “7+3 induction” therapy comprises seven days of cytarabine and three days of daunorubicin, remains the backbone standard therapy for AML, and is often well tolerated [11]. Some cases have also utilized hydroxyurea as a pretreatment for hyperleukocytosis, which has been shown to provide survival benefits in AML induction [12]. Recent trials for newer and more targeted drugs have shown encouraging improvement in clinical outcomes in specific AML populations. However, these interventions also come with the added risk of new adverse events, which may affect the quality of life with limited survival benefits [13]. In the present case, our patient declined further testing and chemotherapy owing to these factors and the added side effects of chemotherapy. Palliative chemotherapy was also offered to the patient; however, she elected to pursue hospice care.

## Conclusions

This case highlights a unique presentation of AMML in the form of cholestasis. Once more common etiologies are ruled out, clinicians should be aware of a broader differential diagnosis of cholestasis, including hematological malignancies. Cytopenias, especially such severe thrombocytopenia below what is typically seen with liver disease, may serve as a diagnostic cue to investigate hematological malignancies. It is imperative to lower the threshold for the diagnosis of AMML-induced cholestasis given the drastic differences in treatment. This case also highlights the poor prognosis of AMML and associated extramedullary manifestations.
